# Assessment of the genetic variability amongst mandarin (*Citrus reticulata* Blanco) accessions in Bhutan using AFLP markers

**DOI:** 10.1186/s12863-015-0198-8

**Published:** 2015-04-18

**Authors:** Kinley Dorji, Chinawat Yapwattanaphun

**Affiliations:** Department of Agriculture, Renewable Natural Resources Research and Development Center, Bajo, Wangduephodrang, Bhutan; Department of Horticulture, Kasetsart University, Bangkok, 10900 Thailand

**Keywords:** Genetic variability, Citrus, Mandarin, Bhutan, AFLP markers

## Abstract

**Background:**

Bhutan is a small Himalayan country lying within the region considered to be the origin of citrus. Diverse citrus wild types grow naturally in different climates, elevations and edaphic conditions, but only mandarin is cultivated commercially. The first report of Huanglongbing (also known as greening disease) in Bhutan in 2003, and the threat it posed to the country’s citrus orchards prompted the collection of mandarin germplasm from across the country. This paper describes the genetic diversity of mandarin accessions in Bhutan using amplified fragment length polymorphic (AFLP) markers.

**Results:**

Twenty three accessions of Bhutanese mandarin were analyzed using AFLP markers to assess the genetic variability that is believed to exist only in Bhutan and some parts of North East India and South China. Five primer pairs (E-ACA/M-CAG, E-ACG/M-CAT, E-ACC/M-CTT, E-AAG/M-CAA and E-ACA/M-CTC) were identified (based on the number and quality of polymorphic bands produced) and used for the analyses. A total of 244 bands were scored visually of which 126 (52%) were polymorphic with an average polymorphism information content of 0.95 per marker. A cluster dendrogram based on multiscale bootstrap sampling categorized twenty three accessions into two broad groups containing eight and 14 accessions, respectively. Group A consisted accessions (*Tsirang1*, *Tsirang3*, *Sarpang1*, *Dagana4*, *Samtse4*, *Dagana1*, and *Trongsa2*) from five districts (Tsirang, Sarpang, Samtse, Dagana and Trongsa) and their grouping was strongly supported by bootstrap analysis (B *p*-value = 96%, AU *p*-value = 86%). Cluster B consisted of 14 accessions divided into three sub-groups (1, 2 and 3). However, bootstrap value supported significantly for subgroup1 (containing accessions: *Tsirang4*, *Sarpang5*, and *Tsirang2*) *and* subgroup3 (with accessions - *Zhemgang2*, *Zhemgang3* and *Zhemgang4*).

**Conclusion:**

This study indicates that Bhutanese mandarin germplasm collected from across the country are genetically diverse although the level of variability differed among the accessions assessed. The variation in genetic variability was observed irrespective of where the accessions were collected suggesting that phenotype and geographical location can serve a basis for future germplasm collection in Bhutan. Further, five primer pair combinations could separate 23 mandarins accessions considered in this study, suggesting that AFLP markers can be a useful tool for future identification.

## Background

Bhutan is a small landlocked Himalayan country between China and India. This region is believed to be the most likely origin of citrus [1], and a rich variability of citrus species exists in the wild, in small back yard farms and in commercially established orchards across Bhutan. Bhutan produces an estimated 50 Gg of citrus fruit annually; over 90% being mandarins. Citrus is grown from as low as 300 meters above sea level (masl) at Sunkosh (27°00’ N, 90°04’ E) in the Dagana district to over 1850 masl at Wengkhar (27°16’ N, 91°16’ E) in the Mongar district.

Mandarin (*Citrus reticulata* Blanco) is believed to be one of the three true *Citrus* species [2]. It is also one of the most diverse group of citrus [2-6]; consisting of numerous intergeneric species and interspecific hybrids [7,8], and as a result is viewed as one of the most challenging with respect to classification and genetic improvement [8]. This genetic variability has been variously attributed to a high proportion of zygotic twins [9], intergeneric cross compatibility, high heterozygosity, nucellar embryony and a long history of cultivation and wide distribution around the world. Most mandarin trees in Bhutan are grown from seed. Local names (*e.g.* “Dorokha local” and “Tsirang local”) applied to mandarins in the different districts suggest variability, and that notion is supported to some extent by morphological studies [10,11]. But the overall genetic variability among cultivated mandarin in Bhutan is unknown.

The sharing of phenotypic characteristics is considered an indication of relatedness. But phenotypic characters only partially reflect the heritable genetic variability because environment also influences growth and development. Recognising the limitation of morphological studies of variability, isozyme analysis has been used in studies of citrus genetic variability [12-15]. However, one of the drawbacks of this technique is that environment and ontogeny may influence the result to some degree. A number of molecular marker-based techniques, differing in their reproducibility and discrimination power, have been used to study genetic variability. Of these, restriction fragment length polymorphism (RFLP) and polymerized chain reaction (PCR)-based RFLP have been used to study phylogenetic relationships within the *Citrus* genus and related genera [16], and to identify interspecific relationships within *Citrus* [17,18]. However, the technique cannot discriminate between closely related genotypes within species [19,20]. Similarly, random amplified polymorphic DNA (RAPD) markers is another useful tool to identify and distinguish citrus species, but reproducibility is low, and the technique cannot identify intraspecific (within species: variety level) variability. Microsatellites, or simple sequence repeats (SSR) markers, a co-dominant and locus specific technique, have proven useful in identifying genetic relationships, but oligonucleotide primer development is expensive and labour intensive.

The AFLP markers approach is a powerful molecular tool used widely in phylogenetics, population genetics, genetic mapping, and cultivar identification. The technique provides highly stable and reproducible information [21] without a need to rely on previous sequence information. AFLP markers have been used in phylogenetic studies of *Citrus* and related genera [22], and homology comparisons within genomes [23]. AFLP marker analysis has also been used in identification of DNA fragments linked with seedlessness in Ponkan mandarin [24], and in the determination of long distance pollen flow in mandarin orchards and its effect on seedless mandarin production [25]. The technique has also been used to link AFLP markers to apomixis genes in pommelo (*C. maxima*) and trifoliate orange (*Citrus trifoliata*) [26]. Thus, the AFLP marker approach is considered a useful tool for cultivar identification and genetic variability studies.

The process of germplasm collection would be more efficient and more likely to result in the collection of genuinely diverse genotypes if it was based on better knowledge of the level of genetic variability present among a population and between populations. Genetic evaluation of accessions from various locations prior to germplasm collection would not only provide information about the geographic distribution of genetic diversity but also help to identify areas of focus for further collection. To date there has been no reports of any assessment based on molecular markers of the genetic variability of mandarins growing in Bhutan. The level of genetic variability remains unknown even for the germplasm already collected.

Therefore, a preliminary population genetic analysis was conducted using the AFLP marker technique to determine the level of genetic polymorphism and variability among 23 mandarin samples that were collected in Bhutan, their selection being based on phenotype and geographic location. The study also assessed the usefulness of AFLP markers to identify mandarins grown in Bhutan.

## Results

### Level of polymorphism

**Figure 1:**
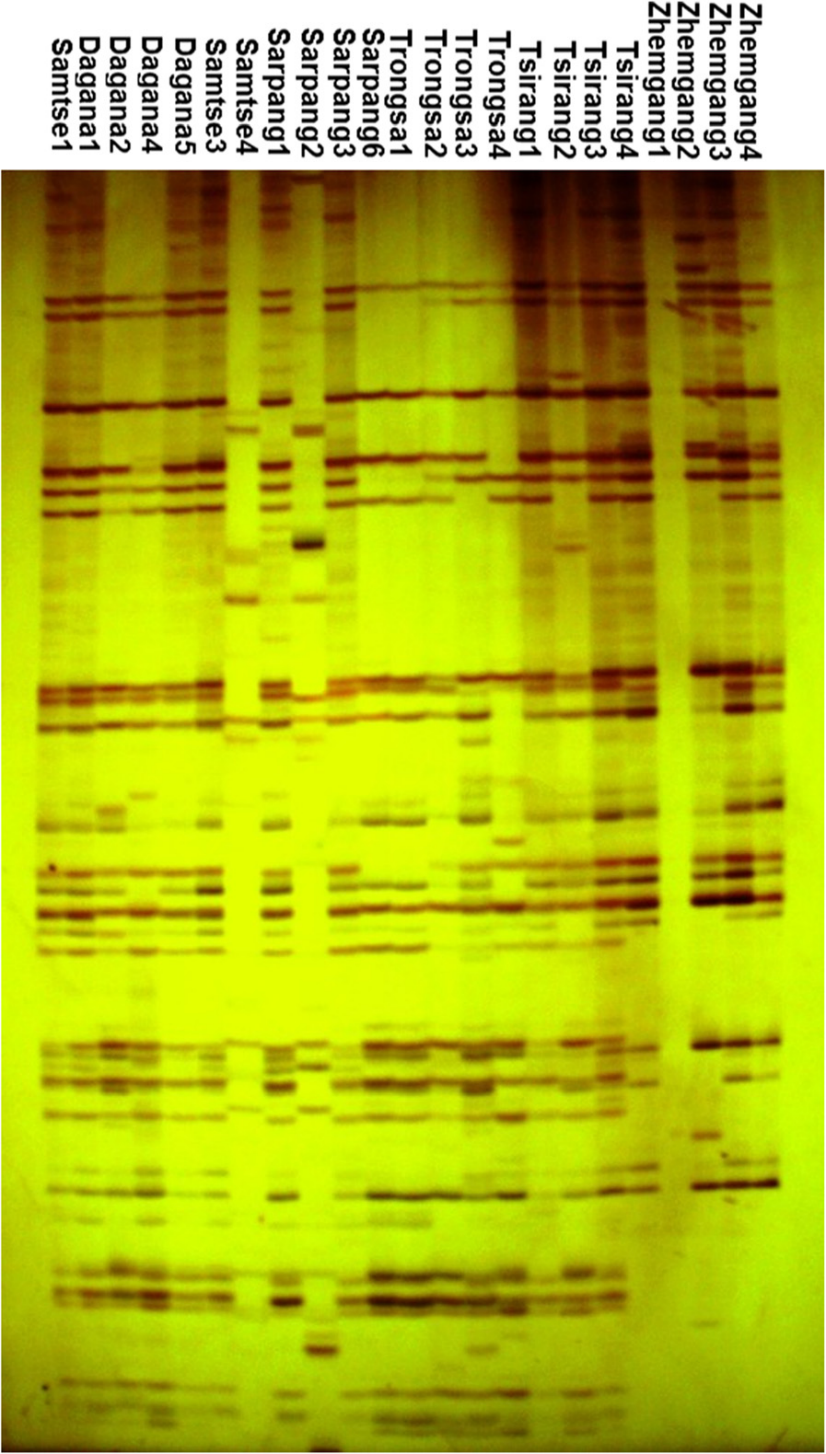
AFLP gel produced by E-ACA/M-CAG primer combination. Each lane corresponds to each accession’s label.

Ten primer pairs were initially evaluated for their discriminating ability and the quality of bands produced. The five different primer combinations and number of polymorphic bands are shown in Table 1. The five AFLP primer combinations of *Eco*R1 and *Mse*1 primer generated a total of 244 bands of which 126 were polymorphic. On average, 52% polymorphism was obtained from each primer combination. The band intensity within a locus also varied among the accessions. The primer pair E-ACA/M-CAG (Figure 1) produced the highest number of scorable bands (84) while the E-AGG/M-CAA primer combination gave the fewest (33). Polymorphism rate ranged from 42% (E-ACA/M-CTC) to 70% (E-AGG/M-CAA).

**Table 1:** **Number of polymorphic AFLP bands observed using 5 AFLP primer combinations**

**Primer combinations**	**Total number of bands**	**Number of polymorphic bands**	**Polymorphism rate (%)**	**PIC**
E-ACA/M-CAG	84	38	45	0.95
E-ACG/M-CAT	53	31	58	0.94
E-ACC/M-CTT	33	17	52	0.94
E-AGG/M-CAA	33	23	70	0.95
E-ACA/M-CTC	41	17	41	0.95
Total	244	126	51

Average PIC per primer pair combination was 0.95. Each of the mandarin accessions used had a unique AFLP fingerprint (banding pattern) that enabled accessions to be discriminated from each other.

### Cluster analysis

**Figure 2:**
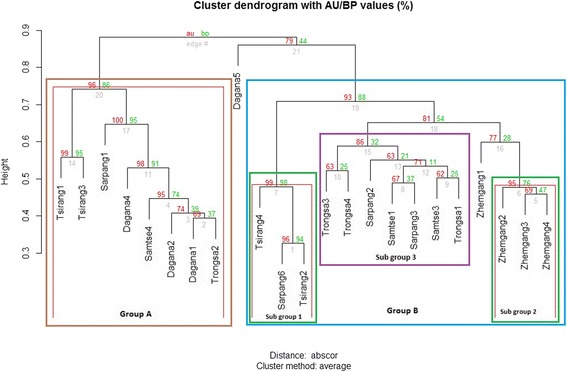
Hierarchical dendrogram as obtained from AFLP data for 23 mandarin accessions with bootstrap values. An inner red lining within boxes shows the group significantly supported by BP/AU p values.

The cluster analysis, represented as a hierarchical dendrogram (Figure 2), separated the 23 accessions into two major groups (A and B). Group B was further divided into three sub-groups (1,2 and 3). However, bootstrap values at (n = 1000) supported significantly (95%) for group A (B *p-*value = 96%, AU *p*-value = 86%) and sub-group 1 (B *p-*value = 98%, AU *p*-value = 98%) and sub-group 2 (B *p-*value = 95%, AU *p-*value = 76%) under group B. Group A contained accessions from all five different locations (Tsirang, Sarpang, Dagana, Samtse and Trongsa). The hierarchical dendrogram suggested a complicated relationship among the mandarin accessions tested. Accessions from Tsirang appeared in both the major groups A and B. Under group B, *Tsirang 2* and *Tsirang 4* along with *Sarpang 6* formed a small clade (subgroup 1). Likewise, *Tsirang 1* and *Tsirang 3* formed a separate clade within Group A. Bootstrap analysis failed to provide evidence of a third cluster under group B.

## Discussion

The range of polymorphism (41–70%) indicated by AFLP analysis suggests the possibility of there being more than one mandarin type, as well as several hybrids, amongst the accessions assessed. The ability of AFLP primer combinations to produce high numbers of polymorphic bands suggests it is a useful tool to identify mandarins grown in Bhutan. This genetic variability and relationship could form a basis for further collection of accessions and genetic improvement strategies. Many species are collectively known as mandarins, but differ in their origin, morphology, distribution and adaptation to the environment [27,28]. Historically, mandarins grown across Bhutan were considered a single variety. Separation of mandarin into two major groups and subsequent divergence to subgroups suggests that Bhutanese mandarin may be better considered to be different biotypes. The probability of Bhutanese mandarin orchards being comprised of clones of a single variety is very low because most of the trees planted by farmers are grown from seeds of diverse and unknown origin. Indeed, Bhutan is described as one of the last citrus fruit producing countries to produce citrus trees from seedlings [29]. Almost all existing mandarin orchards in Bhutan may contain trees that are either zygotic or nucellar in origin in addition to zygotic twins as reported earlier by Das et al. [9]. Because an objective basis for categorising citrus genoptypes into species or varieties based on similarity coefficients is not yet defined, it is difficult to conclude whether Bhutanese mandarins constitute different species or simply genotypes within a single species. This study also supports our earlier description of morphological variability among Bhutanese mandarin [10,11], and provides evidence that variability may have a genetic basis rather than being due exclusively to environmental factors. The balance between genetics and environment in determining phenotype is exemplified by accessions sharing similar morphological characteristics, and originating from the same district, even though there was evidence that they differed genetically.

No geographical affinity was shown between the accessions collected from Trongsa, Tsirang, Dagana, Samtse, or Sarpang. In other words, with the exception of accessions from Zhemgang, accessions from the same district differed genetically. This result is not unsurprising given, as indicated previously, that most mandarin trees in Bhutan are seedlings; being either of gametic or nucellar origin. The genetic variability within districts and between districts is in accordance with the morphological variability reported earlier [11]. The non-uniformity of fruit quality and maturation across the growing regions may be related to genetic heterogeneity as well as geographic and environmental factors [11].

The ability of AFLP to separate closely related accessions supports the findings of Colletta Filho *et al*. [6]. Bhutanese mandarin accessions, all supposedly representing a single variety were well segregated by the technique. The reported high variations among morphological characters of mandarin from different locations in the country [11] is supported by the AFLP analysis, though no linkage between the genetic variability reported here and the morphological variability reported previously can be unequivocally drawn.

An accession from Samtse (*Samtse2*) was genetically similar to an accession from Dagana district (*Dagana4*) despite the distance separating the two districts. This similarity may be due to the trees in each district having been grown from seedlings originating from the National Seed Center, which is the only nursery authorised to supply citrus seedlings. Most of the other accessions were grown from unknown seedling sources. On the other hand, it would seem that the accessions from Tsirang (which has an elevation of 1480 masl) may have resulted from out crossing or might have been driven by environmental factors to adapt. The cluster tree analysis showed limited affinity relating to the accessions’ sources (districts). The arrays of minor groups are difficult to interpret because the dendrogram may have been complicated by homoplasy—a shared character state that is reported to be due either to co-migration of *non*-homologous fragments or the loss of fragments — which can result in an underestimation of genetic variability [30]. Nevertheless, the capacity to separate 23 accessions using five primer combinations shows the potential of AFLP markers to serve as an efficient discriminating tool for characterising mandarin accessions. Although AFLP is a dominant marker approach, the varying intensity of bands or peaks as reported [31] shows that the mandarin accessions analysed comprise a co-dominant and heterozygous population. Further confirmatory study is needed to quantify PCR products. The difference in genetic makeup among accessions from different locations could possibly be ascribed to evolutionary forces to preferentially permit seedling trees with genomes better suited to specific locations to develop through juvenility to reproductive maturity.

## Conclusions

AFLP markers were found to be useful for assessing the extent of genetic variability amongst mandarin accessions collected from across Bhutan. The high level of AFLP polymorphism and variability among the accessions assessed indicate that mandarin types in Bhutan are genetically diverse. The possibility of having duplicate accessions in the mandarin germplasm collection is low, although levels of genetic variation may differ.

## Methods

### Plant materials

**Figure 3:**
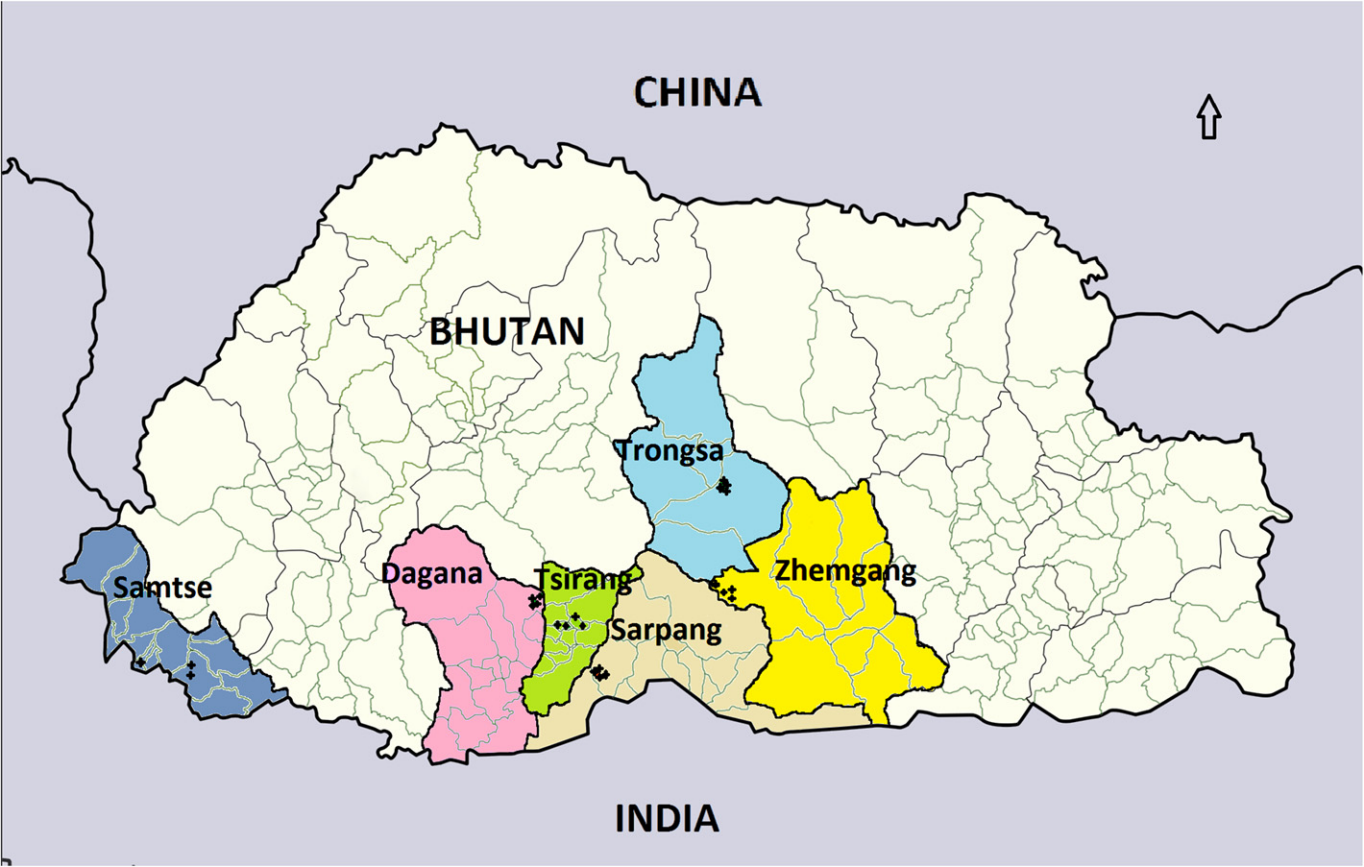
Sampling sites for mandarin accessions in Bhutan. Each accession is indicated by a cross ‘+’ located in map and named after their collection site (eg. Tsirang1 refers to accession1 collected from Tsirang district) and each district highlighted in different color.

The plant material used in this study comprised 23 accessions collected from different districts in the major mandarin growing areas of Bhutan. Semi-hardened green shoots were collected from mandarin trees in 2003, and axillary buds were budded onto seedling rootstocks (Carrizo citrange; *C. sinensis* × *Poncirus trifoliate* L. Raf.) growing in large pots in an insect-proof facility at the Renewable Natural Resources and Development Center (RNRRDC) at Wengkhar. Details of the location for the accessions are shown in (Figure 3). Twenty grams of healthy, fully expanded young leaves were sampled for each accession and stored at −20°C until DNA extraction.

### DNA extraction

DNA was extracted according to the method of Doyle & Doyle [32] with minor modifications. The method is based on the cetyltrimethyl ammonium bromide (CTAB) procedure. Eight grams of fully expanded, healthy young leaves was cut into pieces with sterilized scissors removing the midribs and ground in liquid nitrogen in a mortar with a pestle to a fine powder. The finely ground powder was added to 2% CTAB solution with 60 μl 2-mercaptoethanol, allowed to stand for 30 minutes, interspersed by gently inverting the tube three times every 10 minutes to re-suspend the ground material. Chloroform: isoamylalcohol (24:1) was then added, the tube shaken vigorously for 15 minutes followed by centrifugation at 3000 rpm for 30 minutes. The aqueous supernatant was collected and the process was repeated. An equal volume of ice-cold isopropanol was added to the combined aqueous phases and incubated at −20°C for half an hour. The DNA pellet was collected after centrifugation at 3000 rpm for 20 minutes, rinsed in 75% ethanol, re-centrifuged, air dried and then dissolved in 400 μl tris-EDTA (TE) buffer.

### AFLP analysis

The quantity of the DNA was determined by comparing the extracted DNA with 50 ng lamda (λ) DNA, and quality was judged by the presence of smears in 1% agarose gel electrophoresis. Depending on band size (*i.e.* quantity of DNA), thick DNA bands were re-suspended in 500 to 800 μl TE buffer, and thinner bands were re-suspended in 50 to 300 μl of buffer. Based on earlier studies [8,33-38], only ten primer pairs were chosen and screened for, of which five primer pairs (namely, E-ACA/M-CAG, E-ACG/M-CAT, E-ACC/M-CTT, E-AAG/M-CAA and E-ACA/M-CTC) were used in this study. The AFLP technique was performed as per the protocol described by Vos *et al.* [39] with minor modification. Restriction fragments were produced from the genomic DNA (250 ng) by adding *Eco*RI/*Mse*I (2.5 U each) in a restriction buffer 50 mM (TrisHCl, pH 7.5, 50 mM magnesium acetate, 250 mM potassium acetate) in a final volume of 25 μl. *Eco*RI and *Mse*I adapters were subsequently ligated to the digested DNA fragments. The adapter-ligated DNA (diluted 1:9) was pre-amplified with AFLP primers each having one selective nucleotide using the following cycling parameters: 20 cycles of 30 sec at 94°C, 60 sec at 56°C and 60 sec at 72°C. The pre-amplified DNA was diluted (1:9) based on an assay of the concentration of pre-amplified DNA and the amount needed for band visibility on 6% polyacrylamide gel following electrophoresis. The aliquot was subsequently used for selective amplification with *Eco*RI and *Mse*I primers having three selective nucleotides at the 3’ ends. The cycling parameters for selective amplification were as follows: 1 cycle of 30 sec at 94°C, 30 sec at 65°C and 60 sec at 72°C. The annealing temperature was then lowered by 0.7°C per cycle during the first 12 cycles and then 23 cycles were performed at 94°C for 30 sec, 56°C for 30 sec and 72°C for 60 sec. The reaction products were resolved on 6% polyacrylamide sequencing gels followed by silver staining.

### Data analysis

Information on statistical analysis of AFLP data is scarce. Often the structure and procedures developed for co-dominant markers are applied without considering their appropriateness [40]. Our study adopted a band-based approach; scoring for presence (1) or absence (0) [40]. Bands that resolved poorly on the gel were treated as missing data. Genetic variability was interpreted as the rate of polymorphism (%) and the polymorphism information content (PIC) described by Warburton and Crossa [41]:

$$ \mathrm{PIC} = 1-\varSigma {{\mathrm{p}}_{\mathrm{i}}}^2 $$

where p_i_ is the frequency of the i^th^ allele of individual p.

The data matrix (1 and 0) were subjected to cluster analysis using the “pvclust” package [42] of “R” [43] and following the procedures developed by Shimodaira [44]. The dendrogram generated was grouped and highlighted for highly significant approximately unbiased (AU) p-value and bootstrap probability (Bp) value.

## Abbreviations

A: Adenine
Bp: Base pairs
cm: Centimeter
C: Cytosine
°C: Degree Celsius
DNA: Deoxyribonucleic acid
dNTP: deoxynucleotide triphosphate
DNase: Deoxyribonuclease
dNTP: deoxynucleotide-5/-triphosphate
EDTA: Ethylenediamine tetraacetic acid
EtOH: Ethanol
G: Guanine
g: Gram
H: Hour
HCl: Hydrochloric acid
Λ: Lambda
Ml: milliliter
M: Molar
Mb: Mega base pairs
Mg: miligram
μl: microliter
μM: micromolar
MW: Molecular weight
NaCl: Sodium chloride
NaOAc: Sodium acetate
Ng: Nanogram
PAGE: Polyacrylamide gel electrophoresis
PCR: Polymerase chain reaction
PIC: Polymorphism information content
RFLP: Restriction fragment length polymorphism
RAPD: Random amplified polymorphic DNA
RDC: Research and Development Center
RNA: Ribonucleic acid
RNase: Ribonuclease
Rpm: Rotation per minute
Sec: Second
SSR: Simple Sequence Repeats
TAE: Tris-acetate-EDTA electrophoresis buffer solution
TBE: Tris-borate-EDTA electrophoresis buffer solution
T: Thymine
U: Uracil
